# Surgical Robot for Intraluminal Access: An *Ex Vivo* Feasibility Study

**DOI:** 10.34133/2020/8378025

**Published:** 2020-12-05

**Authors:** Ryu Nakadate, Tsutomu Iwasa, Shinya Onogi, Jumpei Arata, Susumu Oguri, Yasuharu Okamoto, Tomohiko Akahoshi, Masatoshi Eto, Makoto Hashizume

**Affiliations:** ^1^Center for Advanced Medical Innovation, Kyushu University, Japan; ^2^Kitakyushu Municipal Medical Center, Kyushu University, Japan; ^3^Institute of Biomaterials and Bioengineering, Tokyo Medical and Dental University, Japan; ^4^Department of Mechanical Engineering, Kyushu University, Japan; ^5^Department of Advanced Medicine and Innovative Technology, Kyushu University Hospital, Japan; ^6^Kyushu Central Hospital, Japan; ^7^Department of Urology, Kyushu University, Japan; ^8^Kyushu University, Japan

## Abstract

Early-stage gastrointestinal cancer is often treated by endoscopic submucosal dissection (ESD) using a flexible endoscope. Compared with conventional percutaneous surgery, ESD is much less invasive and provides a high quality of life for the patient because it does not require a skin incision, and the organ is preserved. However, the operator must be highly skilled because ESD requires using a flexible endoscope with energy devices, which have limited degrees of freedom. To facilitate easier manipulation of these flexible devices, we developed a surgical robot comprising a flexible endoscope and two articulating instruments. The robotic system is based on a conventional flexible endoscope, and an extrapolated motor unit moves the endoscope in all its degrees of freedom. The instruments are thin enough to allow insertion of two instruments into the endoscope channel, and each instrument has a bending section that allows for up–down, right–left, and forward–backward motion. In this study, we performed an *ex vivo* feasibility evaluation using the proposed robotic system for ESD in a porcine stomach. The procedure was successfully performed by five novice operators without complications. Our findings demonstrated the feasibility of the proposed robotic system and, furthermore, suggest that even operators with limited experience can use this system to perform ESD.

## 1. Introduction

Minimally invasive surgery (MIS) allows surgical techniques that minimize damage to healthy tissues. For example, laparoscopic surgery involves inserting long, rigid instruments (a camera and surgical tools) through a small incision in the patient's skin to access the lesion. This approach results in a smaller incision in a healthy part of the patient compared with conventional open surgery, which usually involves a large incision. MIS offers various benefits to the patient, such as quick recovery and minimal blood loss and scarring. As such, MIS has become a major part of modern surgery. One type of MIS, flexible endoscopic surgery, enables an additional minimally invasive approach for many conditions, such as early-stage gastrointestinal cancer.

Gastrointestinal cancer generally begins from the inner layer of the intestinal wall (*i.e.*, the mucosal layer) then grows toward the outer layer (*i.e.*, to the submucosal layer, then to the muscle layer). If the cancer reaches the muscle layer, a large part of the organ or the entire organ must be removed by laparoscopic or open surgery to mitigate the risk of metastasis. However, if the cancer remains in the mucosal layer, the risk of metastasis is relatively low, and local resection can be performed only around the cancerous tissue. This surgery, called endoscopic submucosal dissection (ESD) [1, 2], can be performed with an intraluminal approach: either *via* the mouth (to the esophagus, stomach, and duodenum) or the anus (to the rectum and colon), using a flexible endoscope. Compared with conventional percutaneous surgery, this surgery is much less invasive (*i.e.*, it does not require an incision in the patient's healthy tissue), and the organ can be preserved, resulting in a higher quality of life for the patient. ESD is popular in Japan and some Asian countries because ESD was originally introduced by Japanese endoscopists. However, in places where ESD is not popular, especially in western countries, patients have fewer treatment options, even for early-stage cancer [3].

One factor that may be hindering the widespread adoption of ESD is that the technique requires a highly skilled operator because it relies on long, thin, flexible instruments that have limited degrees of freedom (DOF) [4, 5]. To facilitate easier manipulation of these flexible instruments, endoscope-based surgical robots are being developed. In contrast to robots designed for laparoscopic surgery, which have been used routinely for many years [6], robotics for flexible endoscopy is a relatively new field. Early studies on such robots initially focused on the application of natural-orifice transluminal endoscopic surgery (NOTES), which was introduced in 2004 [7]. NOTES is a surgical procedure that uses a flexible endoscope instead of a rigid laparoscope; NOTES is mainly used for abdominal organs. Like ESD, access to the lesion is obtained *via* a natural orifice (*e.g.*, the mouth or anus), eliminating the need to make an incision. However, NOTES requires an incision through the gastrointestinal wall to access various organs in the abdominal cavity. Because conventional flexible endoscopes and instruments are not effective in NOTES, several companies are developing multitasking platforms [8–13]. These platforms generally include a flexible endoscope and articulating instruments, such as grasping and cutting devices, and many employ wire transmission and are actuated manually. However, NOTES is still in the experimental phase regarding these developments because this procedure remains technically difficult, even using the new platforms, and most platforms have been discontinued. Recently, several robotic platforms have been proposed, most of which are designed for both the NOTES and ESD procedures.

The Anubiscope [8, 9], which was developed by IRCAD and Karl Storz Endoskope for NOTES and ESD, comprises a custom flexible endoscope and articulating instruments. This manually driven system was later modified by Strasburg University into a motorized version called STRAS [14–17]. The university's researchers implemented visual servoing (*i.e.*, target tracking) [14] and instrument-position measurement [15] in this platform and demonstrated its performance in animal experiments [16, 17]. Endomaster [18–21], developed by Nanyang Technological University in Singapore and Endomaster Pte Ltd., also in Singapore, is a robotic system with a manually driven endoscope that has two instrument channels to accommodate two articulating instruments, each of which has “shoulder” and “elbow” joints rather than uniform bending sections. This system has been demonstrated for both ESD and NOTES in animal models [18, 19], ESD in *in vivo* human experiments [20], and transoral surgery in human cadavers [21]. The Flex robotic system (Medrobotics Corporation, Raynham, MA, USA) [22–25] is a snake-like endoscopic robot. Unlike other endoscopes with single bending sections at the tip, the entire length of this endoscope is flexible; thus, the endoscope can be moved in a follow-the-leader manner by switching two sets of shape-locking sheaths. The system also includes two 4 mm manually driven articulating instruments with continuous bending sections. This robot has been approved by the United States Food and Drug Administration as a medical device, and target clinical applications include intraluminal laryngeal and rectal surgery and percutaneous surgery in the abdominal cavity. However, this endoscope is too short to reach deep sections of the colon. The K-Flex robotic system, developed by the Korea Advanced Institute of Science and Technology in South Korea [26, 27], is equipped with two articulating instruments and a short flexible endoscope. This system has achieved a high payload compared with other proposed systems. Target applications of the K-Flex system include NOTES and percutaneous surgery in the abdominal cavity. Kume et al. [28, 29] proposed a teleoperated colonoscope robot comprising a conventional colonoscope set in a motorized housing that could be controlled using a joystick controller. A unique feature of the proposed system is that it provides force feedback to mimic the resistance force when inserting the colonoscope. The system was used to perform successful colonoscope intubation in a rubber phantom; however, as it was intended only for intubation, the system does not have surgical instruments.

Our group has been developing manually driven flexible endoscopic platforms for ESD [30, 31]. Experimental trials revealed that the endoscope needs to be frequently repositioned to maintain the visual field. Furthermore, both the endoscope and instruments can be teleoperated simultaneously by a single operator, which minimizes the operative cost compared with the current ESD procedure, which requires one clinician and one assistant. Therefore, we developed a motorized version of the flexible endoscopic platform for ESD with two articulating instruments [32]. In this study, we describe the design of the robot and the results of a feasibility study in an *ex vivo* animal model.

## 2. Materials and Methods

### 2.1. Experimental Design

**Figure 1:**
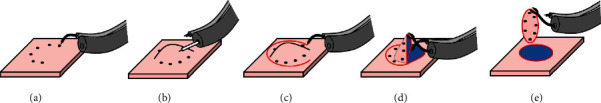
ESD procedure using the proposed robot: (a) marking; (b) injection; (c) circumferential incision; (d) dissection; (e) ESD completion.

This study was a feasibility test of the ESD procedure in isolated porcine stomachs using the prototype robot. The trials were performed by five nonclinicians (medical engineers and managers in a medical company) who did not have previous experience in robotic surgery but had knowledge of ESD. During each operation, an expert clinician who was familiar with the robotic surgery provided verbal instructions to the operator. Each operator began the ESD procedure without training in robotic manipulation. ESD was performed using the same steps as for conventional ESD. Before beginning, a robotic electrosurgical knife and a conventional injection needle were inserted into the additional channel and the endoscope's original channel, respectively. At first, a circle was marked around the simulated lesion using the coagulation mode of the electrosurgical knife (Figure 1(a)). Then, blue liquid was injected into the submucosal layer using the injection needle to create a buffer for dissection (Figure 1(b)). During injection, the injection needle and syringe were held by an assistant. After the assistant changed the device from the injection needle to the robotic grasper, the mucosal layer was circumferentially incised using the electrosurgical knife (Figure 1(c)). Finally, dissection of the submucosal layer was performed using the robotic electrosurgical knife with the help of the robotic grasper (Figure 1(d)). The procedure was completed when the entire lesion was dissected (Figure 1(e)). The entire procedure was recorded on video to analyze the durations of incision and dissection in minutes. Specimen size was estimated from the video in multiples of 5 mm by comparing the thickness of the instrument (2.6 mm) and the specimen. The primary endpoint was ESD completion, and the secondary endpoints were the procedure duration and adverse events.

### 2.2. Mechanical Design of the Prototype Robot

**Figure 2:**
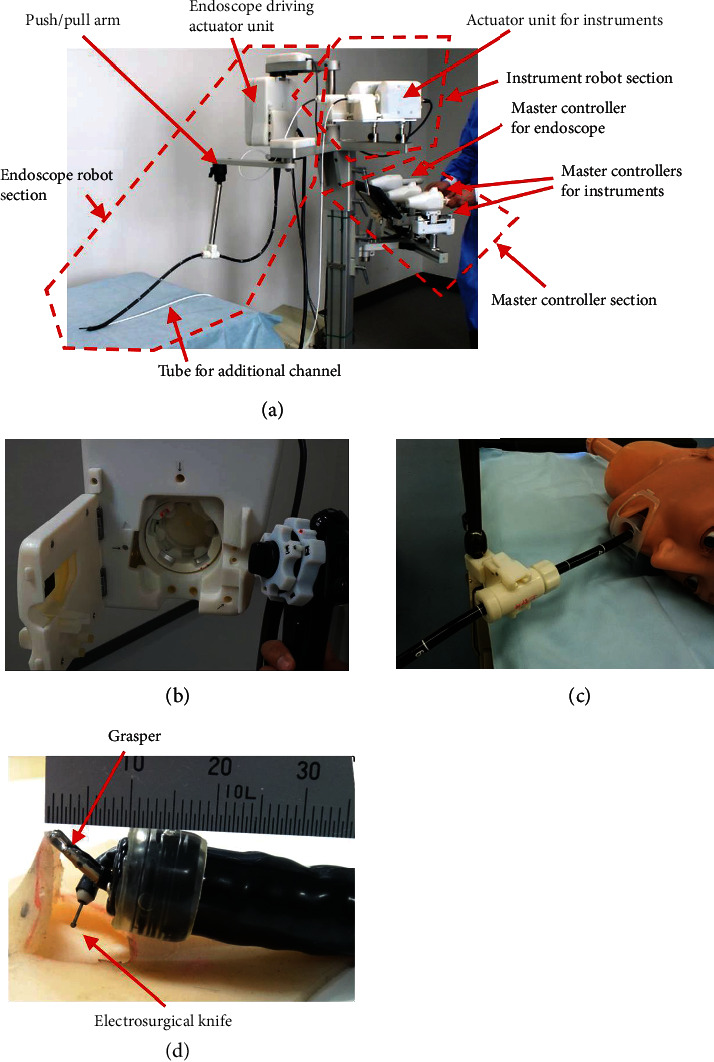
Overview of the proposed robotic system for endoscopic submucosal dissection: (a) photograph of the complete system; (b) magnified view of the endoscope driving actuator unit; (c) magnified view of the coupling for the flexible part of the endoscope; (d) magnified view of the endoscope tip.

Figure 2 shows the proposed robotic system, which is divided into three parts: the robotic endoscope module, the robotic instrument module, and the master controller module. The robot was designed while considering its practical clinical use. First, the system was designed to be compatible with a conventional endoscope without modification. Thus, the healthcare facility can use an existing flexible endoscope and does not need to purchase an additional endoscope for the robot, which can be expensive; this design feature reduces the cost of the system.

The system is equipped with two instruments: a grasper and an electrosurgical knife, and each instrument has a bending section at the tip. The maximum diameter of each instrument is 2.6 mm, which allows the instruments to be inserted into the instrument channels (*φ*: 2.8–3.2 mm) of a commercially available endoscope. This diameter is almost equivalent to the diameter (*φ*: 1.9–2.7 mm) of commercially available instruments that do not have bending sections.

The system was designed to be operated entirely from one console by a single operator. Conventional ESD is typically performed by one doctor and one assistant. If the robot required two clinicians (*e.g.*, one operating the instruments and the other operating the endoscope), this would be costly. Furthermore, all four DOF of the endoscope can be controlled from the console: two dials controlling twisting and inserting the tip of the endoscope and two buttons to control insufflation and suction.

### 2.3. Endoscope Robot Module

**Figure 3:**
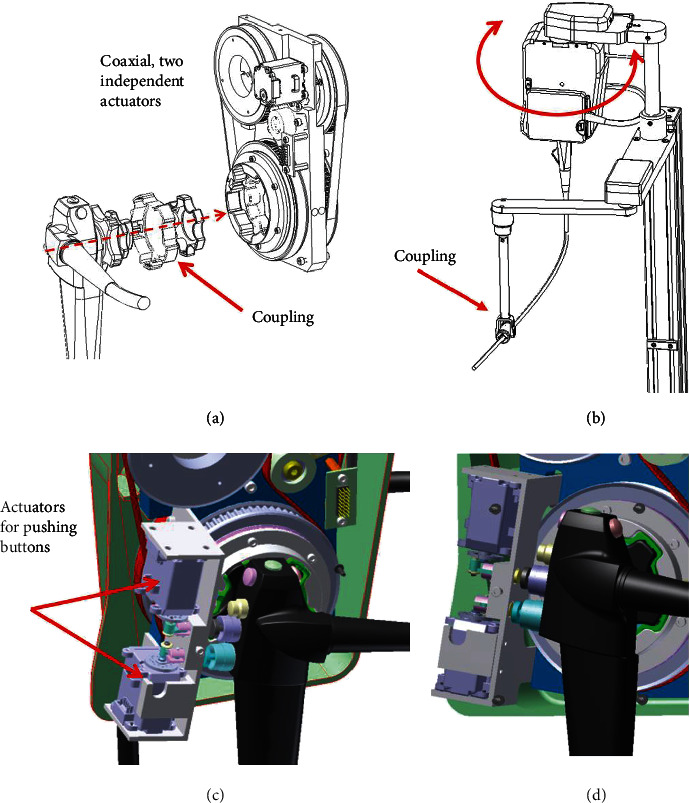
Inside the actuator unit driving the endoscope: (a) the driving mechanism with two dials; (b) twisting motion of the entire endoscope; (c, d) air/suction button actuation mechanism.

The endoscope driving actuator unit is a housing that holds the endoscope grip. A flexible endoscope can be easily set into or removed from this unit by closing or opening the door. The grip of the endoscope has two coaxial angle dials actuating the vertical and horizontal bending. The dials are driven by the two coaxial wheels with fitted couplings (Figure 3(a)). The backlash between the knobs and couplings and between the couplings and actuator unit is not large because the couplings were specially designed so that they fit the shape of the knobs. Each coaxial wheel is driven by timing belts. To actuate the twisting motion of the endoscope tip, the entire endoscope driving actuator unit rotates together with the grip part of the endoscope around the endoscope's shaft, as shown by the arc arrow in Figure 3(b). This motion is transmitted to the endoscope tip because the flexible part of the conventional endoscope is made of a torque-transmissive material. The flexible part of the endoscope is held by a push–pull arm to push or pull the endoscope into or out of the patient's mouth or anus; this motion is similar to a manually operated endoscope. The push–pull arm is coupled with the flexible part of the endoscope by an inner pipe attached to the flexible part of the endoscope and an outer pipe fixed to the push–pull arm (Figure 3(b)). The inner pipe can freely rotate inside the outer pipe; therefore, the coupling transmits the forward–backward motion without interfering with the twisting motion of the endoscope. The stroke range of the forward–backward motion is approximately 15 cm, which is sufficient to move around a lesion but not long enough for insertion from the mouth or anus. Therefore, the endoscope is inserted manually (*i.e.*, without robotic actuation) from the mouth or anus to the lesion, as in conventional endoscopy. When the end of the endoscope is near the lesion, the head of the endoscope is affixed in the robot; after this point, the operator can control all motion from the console. The endoscope driving actuator unit contains two actuators coupled to two push valve buttons to actuate insufflation and suction (Figures 3(c) and 3(d)). Half-push and full-push of the insufflation button control the insufflation and lens washing functions, respectively.

### 2.4. Instrument Robot Module

Two flexible articulating instruments with maximum diameters of 2.6 mm were designed to be inserted into a standard 2.8 mm endoscope channel. Because a standard endoscope has only one channel, a tube was attached to the outside of the endoscope to serve as an additional channel. The instruments can move forward and backward relative to the channel and can be removed from the channel to exchange with other instruments. Each instrument has a 2-DOF bending section at the distal end that employs a continuum mechanism with twelve consecutive hinges. One of the benefits of the continuum mechanism is that the wrist length (*i.e.*, the distance the instrument protrudes from the tip of the channel) is variable. With a fixed-length wrist (*i.e.*, a link-and-hinge mechanism), the instrument can only start to bend after the protruding length exceeds the wrist length. This limitation precludes manipulation close to the camera, which is practically important in flexible endoscopic surgery for several reasons. First, the field of view of conventional endoscopes is very small as they are usually equipped with a wide-angle lens (typically 140 degrees), similar to a fish-eye camera. Therefore, manipulation within 15 mm from the camera is desirable for safe operation. Another reason is stiffness; the instruments are thin and flexible; therefore, if the wrist is long, the moment arm is long, and the stiffness at the tip of the instrument is low, which limits the force that can be applied. The third reason is triangulation; long wrists cause the two instruments to be almost parallel, which is not suitable for precise manipulation. The situation can be understood intuitively as trying to manipulate the instruments using both hands with extended elbows; to manipulate close to the camera, the bending section must have a short bending radius. A laser-cut elastic metal (a nickel–titanium alloy) pipe was used to form a hinge in a previous study [33]; this approach is suitable to make a small-diameter bending section. However, the bending radius cannot be reduced with this design because this would require additional wire force to cope with the elasticity. Thus, the bending section should have as little elasticity and friction as possible.

**Figure 4:**
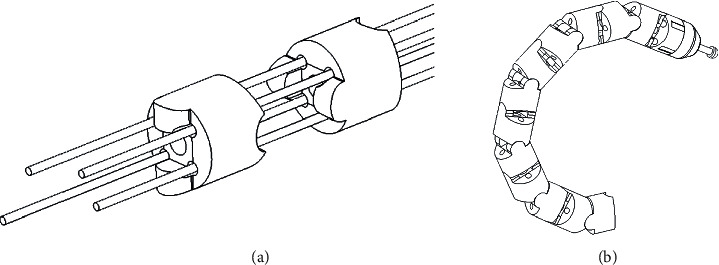
Hinges in the bending section of each instrument: (a) the shape of the hinges and wire routing; (b) bending motion of the hinge unit.

Figure 4 shows the bending section designed for the proposed system. Like a conventional flexible endoscope, the bending section is composed of several consecutive 2-DOF hinges, each of which has two right-angle axes with four wires corresponding to the up–down and right–left bending directions. A unique feature of the bending section is the stacking hinge formed by convex and concave portions, which is achieved only with stacking. A hinge pin is not necessary because the hinges are bound by wires, which helps keep the assembly cost of the bending section low. All hinges are the same shape and made of plastic, which is compatible with mass production and also minimizes the cost. Four wire-guide holes are placed off-axis relative to the hinge to avoid interference between the wire and hinge, which helps create a bending section with a short bending radius. The moving range of the wrist is more than 180 degrees, although a short bending radius is more important than the moving range, as explained earlier.

**Figure 5:**
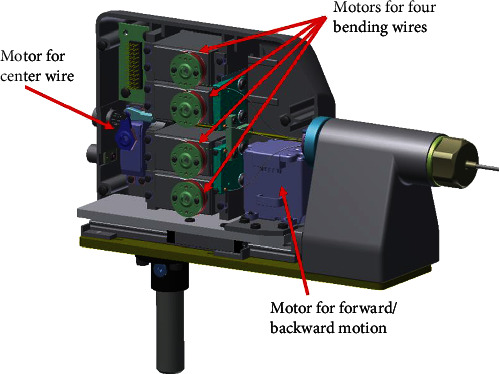
Inside the actuator unit for the instruments.

The bending section is located at the end of the sheath, which is composed of five pairs of Bowden cables covered by a torque-transmissive braided tube and insulating heat-shrink tubing. Four of the five cables are used to control four-way bending, and the center cable is used to actuate the end-effector (*i.e.*, the forceps or the electrosurgical knife). The braided tube cover keeps the instrument from twisting as it is inserted and manipulated, which is necessary to ensure that the controller's direction consistently matches the direction of the bending section. In the motor unit, five actuators drive the five wires. A sixth motor actuates the movement of the entire motor unit relative to the instrument channel to control the forward–backward motion. For the prototype intended for this feasibility study, we used an R/C servo for rapid prototyping, as shown in Table 1. The proximal ends of the wires are connected to the pulleys, which are directly attached to the output shafts of the motors, as shown in Figure 5.

### 2.5. Master Controller Module

**Figure 6:**
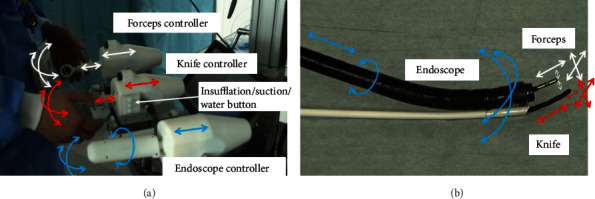
Master controller and control axes: (a) master controller and its control axes; (b) corresponding moving axes of the robot.

The master controller for each flexible articulating instrument has a 2-DOF joystick controlling up–down and right–left motion. The joystick is mounted on a linear guide to allow forward–backward motion input. The operator can push or pull a knob on the grip using his or her thumb to control the open–close motion of the grasper jaw or to adjust the length of the electrode. The master controller for the endoscope is similar, but the controller has a twisting axis in the grip to control the twisting motion of the endoscope and does not have a knob. All sensors are rotary or linear potentiometers. Three buttons on the master controller control the suction, insufflation, and lens washing functions. Joint-to-joint control is implemented in the control program. The parameters and specifications are shown in Table 1; Figure 6 shows the details of the master controller and control axes.

## 3. Results

**Figure 7:**
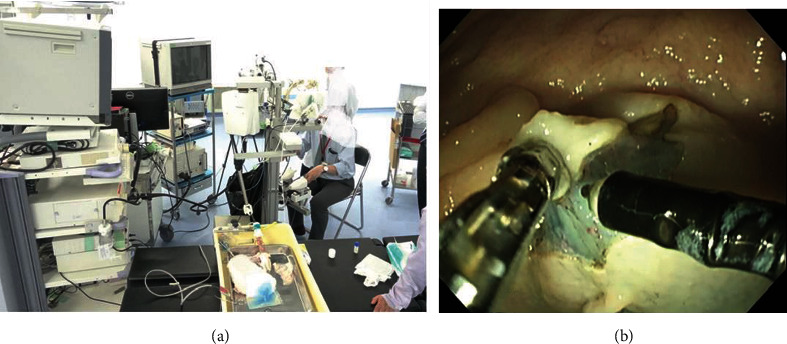
(a) Experimental setup; (b) endoscopic view during an ESD procedure using the proposed robotic system.

Five ESD trials were performed by five participants, and all ESD procedures were successfully completed with en bloc resection. The procedure times and specimen sizes are shown in Table 2. The specimen sizes ranged from 10 mm to 25 mm, and the mean ± standard deviation (SD) was 16.0 ± 5.5 mm. The means ± SDs of the circumferential incision time and dissection time were 11.2 ± 8.6 minutes and 12.4 ± 5.5 minutes, respectively. Dissection speeds per area calculated using the formula area = *π*(specimen size)2/4 ranged from 3.4 min/cm^2^ to 15.3 min/cm^2^. No adverse events were observed.  Figure 7 shows the experimental setup and an endoscopic view during ESD.

## 4. Discussion

Our trials in *ex vivo* animal stomachs showed that ESD using the proposed robot system was feasible. Notably, each operator performed ESD for the first time, and all operators were novices in endoscopy. These findings suggest that the proposed system will significantly reduce the technical hurdles associated with ESD. In our previous study [30], the average dissection speed of ESD endoscopists was 2.7 min/cm^2^. In this study, ESD was performed only once by each participant. Thus, although these procedure times are relatively slow compared with those generally achieved in clinical practice, we expect that the procedure could be completed more quickly as the user gains experience with the robotic system. We observed large differences in operation times between cases. The possible reason is that some operators were generally good at machine handling and others were not. However, further study is required to clarify this issue.

Our robotic system was designed with a focus on clinical adoption. This system is compatible with a conventional endoscope, to minimize the added cost of the system. The instruments are thin enough to be inserted into the 2.8 mm channel of the endoscope, and only one clinician is required to operate both the endoscope and instruments. Therefore, the personnel expense associated with the procedure is minimized. No previously developed robotic system offers all of these features.

This study had several limitations: First, we did not consider sterilization of the system. For the commercial version of the system, the instruments will be disposable. Furthermore, mechanical connectors between the instruments and the motor unit and a mechanism to ensure biological separation between the endoscope and endoscope-driving unit might be required; these features are not present in the current prototype. Finally, the participants were not clinicians, and their number was limited. We intend to address these limitations in a future study.

On the basis of the experimental results, we conclude that this novel robotic system can be used to safely perform ESD in an *ex vivo* porcine stomach. Our findings suggest that even operators who have limited experience can use the system to perform ESD.

## Figures and Tables

**Table 1:** Specifications of the proposed robotic system.

Parameter	Specification
Flexible endoscope used for experiment	GIF-Q260J (Olympus Medical Systems, Japan)

Instruments	Diameter *φ*2.6, length 2.2 m (1) Electrosurgical knife, needle shape (2) Grasping forceps

Endoscope robot DOF	Bending 2 + forward/backward 1 + twisting 1 + air/water button 1 + suction button 1 = 6 DOF

Instrument robot DOF	Bending 2 + forward/backward 1 + open/close forceps 1 = 4 DOF

Master DOF	<For endoscope> Joystick up‐down 1 + right‐left 1 + forward‐backward 1 + twist 1 = 4 DOF (sensors: potentiometers) Push switch 3 buttons (air/water/suction) <For instruments> Joystick up‐down 1 + right‐left 1 + forward‐backward 1 + open/close forceps 1 = 4 DOF (sensors: potentiometers)

Control algorithm	Joint-to-joint control

Motors	<Endoscope robot> RS406CB × 2 (dial rotation) RS406CB × 1 (push/pull) RS406CB × 1 (twisting) RS406CB × 2 (air/suction button) <Instrument robot> BLS177SV × 4 (wire for bending) BLS177SV × 1 (wire for forceps) RS406CB × 1 (forward/backward) (All from Futaba Corporation, Japan)

Microcontroller unit (MCU)	Microcontroller board based on STM32F103 (STMicroelectronics, Switzerland) connected with PC via USB2.0

PC	Core2 duo, Windows

Circuit	Potentiometers/switches---MCU1---PC---MCU2---motors

**Table 2:** Experimental outcomes.

Case #	#1	#2	#3	#4	#5
Duration of incision (minutes)	26	7	8	4	11
Duration of dissection (minutes)	18	8	12	18	6
Total (minutes)	44	15	20	22	17
Estimated specimen size (mm)	25	15	10	15	15
Dissection speed per area (min/cm^2^)	3.7	4.5	15.3	10.2	3.4
